# Composition and influencing factors of professionals’ capacity in public health emergency rescues: a qualitative study

**DOI:** 10.3389/fpubh.2024.1338839

**Published:** 2024-05-09

**Authors:** Fangfang Zhong, Yin Lin, Ying Chen, Yazhuo Gao, Xuehua Zhu

**Affiliations:** School of Nursing, Zhejiang Chinese Medical University, Hangzhou, China

**Keywords:** professional, public health emergencies, emergency rescue, ability composition, influencing factor, qualitative research

## Abstract

**Objective:**

To explore the composition and influencing factors of professionals’ capacity in public health emergency rescues.

**Methods:**

A descriptive qualitative design was used in this study. Medical workers, managers, and members of an emergency rescue team in Hangzhou, Zhejiang, were recruited for participation through a purposive sampling method. The data were collected using semi-structured interviews and analyzed using a conventional content analysis method.

**Findings:**

A total of 2 themes and 13 sub-themes emerged from the analysis: ability composition (knowledge reserve, early warning assessment, information reporting, emergency response, self-protection, personal ability, coordination and cooperation, health education) and influencing factors (educational background, region, experience, hospital level, human resources, and financial investment).

**Conclusion:**

These findings offer a basis for the construction of a related indicator system and provide a reference for relevant departments to further optimize their emergency education and training, strengthen their emergency drills, and improve their emergency rescue abilities. The findings indicate that it is necessary to pay attention to the construction of an emergency rescue team, adjust the ratio of personnel, improve their remuneration, and promote work enthusiasm to improve the emergency rescue ability of an organization.

## Introduction

A public health emergency refers to a sudden surge in major infectious diseases, mass unexplained diseases, significant food poisoning or occupational poisoning, and other events that seriously affect public health (1). Since the beginning of the 21st century, with economic development and rapid population growth, several major public health emergencies, including SARS, Ebola virus, COVID-19, and others, have occurred worldwide (1–3). The sudden and unpredictable nature of public health emergencies (4) can significantly affect human lives, property, safety, and physical and mental health. Furthermore, it can also jeopardize social and economic development and national security (5, 6).

Emergency rescues form an integral part of emergency management. Emergency rescues encompass emergency responses, emergency rescues, medical treatments, logistics, and other activities to protect the lives and properties of individuals and maintain social order and public safety in the case of natural disasters, accidents, public health incidents, social security incidents, and other emergencies (7). Medical personnel, rescue teams, and other professionals are the driving forces behind clinical frontline rescues, and their emergency rescue abilities directly affect the smooth operations of on-site rescuers and the safety of patients (8). One study demonstrated that the emergency rescue capacities of medical personnel, public health personnel, and other relevant professionals in public health emergencies were not high, especially in primary medical departments (9–11). The objective of this study was to explore the composition and factors affecting the capacity of professionals in public health emergency rescues. It was hoped that the findings would provide a theoretical basis for constructing an emergency rescue competence index system for professionals dealing with public health emergencies and developing relevant continuing education and training.

## Methods

### Design and sample

A descriptive qualitative design was used in this study. Such an approach avoids preconceived categories and allows the researchers to immerse themselves in the data, generating novel insights and providing a richer understanding of phenomena (12). A targeted sampling method was used in this study to recruit clinical medical workers, community medical workers, and rescue team members from Hangzhou, Zhejiang. The recruitment of a sample with maximum variation (gender, age, education level, and position) was facilitated using the targeted sampling method. The inclusion criteria for this study included: (1) in-service clinical medical workers, community medical workers, and rescue team members aged between 18 and 60 years; (2) over 5 years of work experience in this field; and (3) a good language communication ability and voluntarily agrees to participate in the study. The exclusion criteria were: (1) unable to cooperate with the investigator due to physical conditions.

### Participant characteristics

After data saturation, the final sample comprised 15 clinical medical workers, community medical workers, and rescue team members. Six participants were male, and nine were female, with an age range of 28 to 54 years. Detailed demographic data are shown in Table 1.

**Table 1:** Detailed demographic data of the participants.

Participant	Age (years)	Gender	Professional Title	Duty	Working years	Education	Emergency rescue experience
1	42	Female	Nurse-in-charge	Vice Head Nurse	20	Master’s degree	Yes
2	40	Male	Assistant director physician	Head of health section	11	Master’s degree	Yes
3	49	Male	Assistant director physician	None	28	Bachelor’s degree	Yes
4	47	Female	Associate senior nurse	Head Nurse	29	Bachelor’s degree	Yes
5	28	Female	Nurse practitioner	None	6	Bachelor’s degree	No
6	48	Female	Associate senior nurse	None	31	Bachelor’s degree	Yes
7	30	Female	Nurse practitioner	None	7	Bachelor’s degree	Yes
8	38	Female	Nurse-in-charge	None	17	Bachelor’s degree	Yes
9	29	Female	Physician-in-charge	None	4	Master’s degree	No
10	35	Female	Physician-in-charge	None	9	Doctor	Yes
11	31	Male	Nurse-in-charge	None	8	Bachelor’s degree	Yes
12	54	Female	Assistant director physician	Head of the health service	32	Bachelor’s degree	Yes
13	29	Male	Nurse practitioner	None	6	Bachelor’s degree	No
14	31	Male	Nurse-in-charge	None	8	Bachelor’s degree	Yes
15	36	Male	Rescue worker	None	13	Bachelor’s degree	Yes

### Interview outline

Data were collected using semi-structured interviews that were conducted face-to-face. The interview guide was developed based on a literature review. The interviews were focused on the following seven questions: (1) Do you know what a public health emergency is? (2) What do you think “emergency rescue” is? (3) What public health emergencies have you experienced in your career? Can you tell me something about your experience and how you participated in the emergency rescue? (4) How did you participate in the emergency response during the last COVID-19 pandemic? What abilities do these jobs require of professionals? Can you elaborate on this? (5) What roles and responsibilities do professionals play in the course of public health emergencies (before, during, and after)? (6) In your opinion, what abilities and qualities must professionals possess during different types of public health emergencies (such as infectious diseases, food poisoning, occupational poisoning, accidents, and leakage)? Could you give specific examples of behaviors that reflect these abilities? (7) What factors do you think will affect the emergency rescue abilities of professionals? What can you suggest to improve the emergency rescue abilities of professionals?

### Data collection methods

The research team consisted of nursing postgraduate students and an associate professor both of whom have extensive experience in conducting qualitative research studies. To protect the privacy of the interviewees and avoid their concerns, the interviews were conducted in a quiet, independent room or a conference room at the interviewees’ workplace. Before the formal interviews, the researcher (ZFF) communicated fully with the interviewees, informed them of the purpose and content of the interviews, and audio-recorded the entire interview after obtaining their consent. They were also told that the relevant data would only be used for statistical analysis and that the researcher would maintain strict confidentiality. Before the interviews, the interviewer did not establish relationships with the participants prior to the interviews. The interviewer mastered the interview outline and adjusted the order and content according to the interviewees’ answers but kept it related to the topic. The non-verbal expressions of the interviewees were focused on and recorded at any time, and the follow-up questions were asked promptly. Each interview lasted 30–40 min.

### Data analysis

Data collection and analysis were performed simultaneously. The traditional content analysis method was used to analyze the qualitative data. The traditional content analysis research begins with observation, and the coding is defined in the data analysis, and the coding comes from the data. It has the advantage of obtaining information directly from the interviewees without imposing the researcher’s point of view (12). The specific steps were as follows (13): (1) Immediately after each interview, the researcher (ZFF) transcribed the recording verbatim and compared it with the original recording to ensure accuracy. According to P1 ~ P15, the interview records of each interviewee were numbered, and their independent Word documents were established. The emotional changes and action expressions of the interviewees during the interview were marked in the corresponding position, and a researcher (CY) was invited to proofread the text and recording content. Two researchers (ZFF and LY) repeatedly read the text to understand the participants’ ability to respond to public health emergencies. (2) Two researchers (ZFF and LY) independently encoded the essential and recurring words and phrases (units of meaning) in the text, and the code of similar content formed a sub-theme. (3) The sub-topics were jointly summarized by two researchers (ZFF and LY) to form a theme. Some representative examples were excerpted from the qualitative data. (4) All members of the research team reached a consensus on the research topic and discussed whether the research topic had reached theoretical saturation, which means no new findings or themes occurred from an analysis of newly collected materials. The NVIVO11.0 software was used to store and manage the qualitative data obtained.

### Rigour and reflexivity

First, the researcher received systematic training in qualitative research, with proficiency in interviewing skills. The interviewer was supervised by an associate professor proficient in qualitative research methods. Second, the interviewer encountered unclear or questionable information during the interview, and asked or recounted the interviewee several times in time. Third, all transcripts were returned to the interviewees to validate the interview content. The actual information was verified to ensure the authenticity, integrity, and accuracy of the data. While encoding data, the researchers held a neutral position and did not bring their values and professional identity. The data were independently analyzed by two researchers. Finally, the final coding and theme were determined from the discussion of the research team members. This reporting was guided by the Comprehensive Standard for Reporting Qualitative Research (COREQ) (14).

### Ethical considerations

This study was approved by the Ethical Review Board (ERB) of the Zhejiang Chinese Medical University (DSRB-Ref 20200529-1).

## Findings

Based on the qualitative data, 2 themes and 13 sub-themes about the composition and factors that affect the capacity of professionals were developed.

### Theme1: the emergency rescue ability of professionals in public health emergencies

#### Knowledge reserve ability

Solid theoretical knowledge is essential for emergency rescues. All participants mentioned that participating in an emergency rescue requires a rich reserve of knowledge. Some participants reported that in addition to relevant first aid knowledge, professionals should also deeply understand public health emergencies, emergency rescues, emergency plans, and related laws and regulations. Such knowledge expectations are consistent with the public health personnel competency standards proposed by Columbia University in the United States (15). Notably, the interviewees were asked, “What do you think of your own level of emergency rescue capability?” and all respondents agreed that their actual emergency first aid skills and first aid knowledge were far from sufficient.

“Professional knowledge is needed. In my opinion, only with such specialized knowledge can we judge and handle emergencies. Theoretical knowledge is the foundation of all abilities and skills” (P9).

“Just like the recent COVID-19 outbreak, because we have a clear understanding of the emergency planning and processing processes, we can know exactly what to do next” (P12).

#### Early warning assessment capability

Rogers and other experts (16) argue that in addition to mastering relevant disease knowledge and clinical skills, professional medical staff also need to be able to apply their skills in areas such as monitoring potential hazards and risk information transmission. They must also be able to analyze the risk factors in the environment to prevent incidents or further deterioration. The views of some of the respondents of this study were consistent with this assertion. Specifically, participants mentioned that professionals should be sensitive to emergencies. That is, they must have a developed early warning assessment capability, which is necessary for professionals to participate in emergency rescue in public health emergencies. In addition, some respondents reported that early identification, judgment of abnormal information that may occur in public health emergencies, and investigation of possible risks can reduce the occurrence of emergencies and their possible harm and loss to a certain extent.

“For example, in our nearby school, several students in the same class came to see the doctor because of diarrhea one after another. In this case, I think as a physician, you should be aware that it could be mass food poisoning, and that requires us to have an early warning and assessment capability” (P3).

“As a professional we should be sensitive and be able to recognize that such events may be public health emergencies…” (P10).

#### Information reporting capability

Timely information reporting, standardized reporting processes, and accurate reporting content help minimize the damage caused by public health emergencies (17). Respondents reported that medical staff should be able to report, collect, and analyze information, including knowledge of reporting requirements, the registration process for public health emergencies, and the legal reporting time limit for infectious diseases. However, nine respondents indicated that their awareness of reporting was not high, which may increase the risks associated with potential emergencies. The research evidence shows a clear relationship between the reporting ability of medical institutions and employees’ sense of responsibility and training (18). Therefore, improved training and education may be an effective way to improve the information reporting capabilities of professionals.

“A standardized reporting process is essential, and only when a health care worker reports such an unforeseen situation does it draw the attention of others to the situation and somehow mitigates its adverse effects…” (P13).

#### Emergency response capability

All participants mentioned that emergency response capabilities such as emergency rescue, injury classification, transportation of patients, the use of general rescue techniques, and emergency equipment capabilities are necessary for professionals. Patients at the scene of emergencies often have complex injuries, and professionals need to be good at observing changes in the condition of patients and dealing with them in a timely manner. When there is an emergency situation, professionals must respond quickly, make timely judgements, and take appropriate measures to treat the wounded. Studies have shown that medical staff have a good grasp of fundamental basic first-aid techniques, However, their first-aid capabilities for rare public health emergencies are insufficient (19, 20), which is consistent with the views of some respondents in the current study. Therefore, in addition to mastering general first-aid skills such as cardiopulmonary resuscitation, medical staff also need to master first-aid capabilities for infectious diseases, occupational poisoning, and chemical leaks (21).

“Master the first-aid technology. Like this COVID-19 pneumonia, the risk rate of this disease is much higher than that of normal diseases. We should be ready to give patients tracheal intubation and CPR cardiopulmonary resuscitation at any time…” (P14).

“For critically ill patients, we also need to have knowledge of emergency equipment such as ECMO, artificial livers, and artificial kidneys” (P8).

#### Self-protection capability

In the “State Nursing Directors Association Opinion” issued for disaster emergency response missions, the U.S. Public Health Preparedness Commission proposed that the capabilities required by medical personnel also include personal protection (22). Self-protection and management of hospital-acquired infections is one of the critical steps in controlling the spread of public health emergencies, especially during major infectious disease outbreaks (23). The on-site environment of an emergency rescue is usually an exceptional environment that is complex and changeable. Rescuers often face problems such as a poor working environment and high work intensity. Emergency rescuers are three times more likely to be injured or killed than ordinary staff (24). All respondents in the current study experienced the COVID-19 epidemic. Thirteen respondents indicated that they needed to do a good job of self-protection during this time, such as strict standardization of the use of personal protective equipment and strictly abiding by the disinfection and isolation protocol so as to prevent self-infection and cross-infection.

“First of all, we should take protective measures for ourselves and adopt standard precautions. All patients should be considered infected and screened in isolation at specific times” (P15).

“Self-protection and prevention of hospital-acquired infections is essential. For example, we must be able to skillfully put on and take off protective clothing and wash our hands. Then, hand hygiene must be maintained before contact with the patient to avoid cross-infection” (P8).

#### Personal capability

Individual capabilities are seen as the cornerstone of the entire emergency response capability system (25). Participants reported that professionals should respond to public health emergencies with a conscientious and responsible attitude, good mental and physical fitness, and a spirit of saving lives and helping with injuries (26, 27). Medicine is an ever-developing discipline, and professionals need to commit to active learning and regular participation in emergency drills and courses related to emergency management. In addition, medical staff cannot ignore their own psychological adjustment while continuously improving their core emergency response capability for public health events (28). Notably, in the current study, when asked about their psychological state in the novel COVID-19 epidemic, most respondents reported that they had felt anxious and confused, which is consistent with previous findings (29). Therefore, as the main force behind rescue work, medical staff need to undergo timely adjustment of their psychological state to adapt to the special working environment during public health emergencies and invest in emergency rescue work as soon as possible.

“Public health emergencies are inherently high-risk. We must be psychologically prepared and dedicated…” (P10).

“In addition, for individuals, we need to strengthen the relevant learning. Such public health emergencies will not happen often, but we need to participate in as many emergency drills as possible to improve our emergency response capabilities” (P2).

#### Coordination and cooperation capability

Several studies have shown that good coordination and cooperation can ensure the smooth running of emergency rescues and improve rescue efficiency (30, 31). Communication ability in emergencies not only refers to communication with patients and family members but also includes reporting information to relevant government agencies and communication with news media and the public. In addition, in the process of an emergency rescue, professionals must cooperate with other staff and adhere to the allocation of personnel and materials by each unit. In our survey, respondents agreed that public health emergencies are often sudden, and personnel and materials are often temporarily allocated. Such circumstances requires the relevant personnel to have the good ability to coordinate and cooperate, follow the distribution, and rationally use emergency materials.

“Epidemics are often unpredictable. Manpower and material resources are very scarce at these times. As a member of the medical profession, we must first and foremost be on call 24 h a day, obey our superiors, and follow orders” (P13).

“Close cooperation with relevant line ministries, including the Government, or rescue forces” (P15).

#### Health education capability

Health education is the most effective and economical way to mobilize social forces, raise public health awareness, reduce the risk of infection, and enhance information disclosure (32). Several studies have emphasized the importance of communication in connecting with the public during a public health emergency (33–35). Such views are consistent with the views of some respondents in the current study. Seven respondents reported that educating community residents on how to protect themselves and how to respond to emergencies is also part of emergency rescue work. Additionally, respondents believed that during public health emergencies, the management of the health status of community residents, especially the management of special populations, such as the older adult with chronic diseases, should not be ignored.

“For example, in the case of this new type of coronavirus pneumonia, health education will enable residents to know how to protect themselves” (P5).

“Some people do not know much about respiratory transmission, and health education will let them know what the routes of infection are, so that they can take some precautions in advance” (P10).

### Theme 2: influencing factors

#### Educational background

All respondents expressed that educational background will affect the emergency rescue abilities of professionals, consistent with the results of Yang et al. (36) and Ren et al. (37). The occurrence of public health emergencies is often unpredictable, and the emergency response process involves a complex rescue environment and heavy rescue tasks. Such characteristics require professionals to not only have a comprehensive professional theoretical base and sufficient skills, but also an extensive knowledge base on epidemiology, public health protection, psychological assistance, and other aspects. Compared with medical staff with low educational backgrounds, medical staff with high educational backgrounds may have received more systematic emergency rescue professional training, resulting in better-developed knowledge and mastery. In addition, highly educated professionals have more awareness of and ability to engage in autonomous learning; they accept new things faster, which can contribute to an improved emergency rescue ability in public health emergencies. Accordingly, health management departments should rationally allocate human resources, increase the training of highly educated talents, and meet the needs of community healthcare (38).

“I think that educational background also affects the ability to respond to emergencies. More educated people are more able to accept new things and have a stronger sense of active learning, so their emergency response capability will be better” (P3).

#### Region

Participants reported that there would be substantial differences in the level of first aid provided between different regions, and the ability of professionals would certainly be different. Evidence suggests that the higher the level of a medical institution, the stronger its medical staff’s health emergency response capacity (39). Ma (40) described several reasons for the weak health emergency management ability of primary medical institutions. First, they lack emergency preparedness; for example, they lack emergency material reserves for the prevention and control of the COVID-19 epidemic. Second, their emergency response capacity is not strong; they lack sufficient information support and modern science and technology. Third, due to economic and human resource constraints, a lack of professional talents and rescue equipment and facilities at the grass-roots level has reduced the emergency rescue capacities of these institutions. Therefore, coordinated emergency management and regional development should be strengthened by enhancing communication and interaction between provinces and cities.

“The provincial hospitals in our city have no problem with this, while the lower-level hospitals may not do it well enough. Because our hospitals are often faced with public health emergencies, such as the previous bird flu, H7N9, and SARS, our hospitals are more experienced in emergency care in this area, and lower-level hospitals may not do so well” (P1).

“There is a great difference in the level of medical care between urban and rural areas, and the competence of professionals is certainly different” (P7).

#### Experience

Studies have shown that a larger number of emergency response experiences predicts a higher self-evaluation of coping capacity (41, 42). In the current study, thirteen respondents stressed the important role of practical experience in improving emergency response capabilities. They expressed that experience is the best teacher, and experienced medical staff can better combine theoretical knowledge with practice and summarize their own shortcomings and deficiencies during the rescue process, enabling them to strengthen these factors in later work. Such an approach is consistent with the “novice to expert” theory proposed by American scholar Benner. This theory holds that medicine is an applied discipline that requires both theoretical knowledge and practical application. With the gradual accumulation of work experience, knowledge gradually develops from shallow and explicit to deep and implicit, and an individual’s professional ability gradually develops and improves (43, 44). With an increase in working years, medical staff obtain richer working experiences, including experience in emergency and disaster management, and this translates to higher emergency rescue abilities (45).

“I think one’s personal clinical experience affects one’s rescue ability, and when there is an outbreak, we need to determine if it is likely to be a public health emergency, such as food poisoning” (P6).

“The more experience you have, the better your overall competence in all areas will be, compared to someone who has just graduated or has not experienced such activities” (P10).

#### Hospital level

Participants expressed that whether a hospital focuses on training in emergency response capabilities, whether relevant emergency response plans exist, and whether the stockpile of materials is adequate all affect the emergency response capabilities of professionals, which is consistent with other research results (46, 47). The higher the level of the hospital, the more emphasis on the cultivation of core emergency capabilities; accordingly, more training opportunities related to major infectious diseases are provided and this strengthens the ability of professionals to carry out practical exercises. Studies (48, 49) have indicated that the emergency rescue ability of professionals who have participated in emergency drills is generally higher than that of those who have not participated in relevant drills. In a systematic evaluation of the disaster preparedness of professionals (50), the importance of simulation exercises for improving emergency rescue capabilities was also emphasized. On the other hand, high-level hospitals admit more acute, severe, and complex patients on weekdays, and the medical staff have relatively more treatment experience (47). Such interpretation is consistent with the above findings suggesting that the experience of professionals affects their rescue abilities.

“The organizational ability and coordination ability of the hospital will have an impact. Our medical personnel are only a small part of the emergency rescue of public health emergencies. Well-organized hospitals and well-coordinated departments can help us to be more effective” (P10).

“Does the hospital have corresponding systems and processes for various public health emergencies? Any training, any drills? Is there a special agency in charge of this? I think these have a great influence on our professional emergency rescue ability level” (P1).

#### Insufficient human and financial investment

Factors such as a lack of emergency professionals, low financial investment, and a lack of attention from the relevant authorities will affect the enthusiasm of professionals to a certain extent, and this will, in turn, affect their emergency rescue abilities (51). In particular, when public health emergencies occur in grassroots medical institutions, the rescue capability of the institution is directly related to the quality and timeliness of its emergency response (40). Most respondents in the current study reported that the hospitals they work in invest little in emergency rescue, and professionals’ efforts in emergency rescue have not been remunerated accordingly. When a public health event occurs, an emergency team is temporarily established, and often, there are not enough human resources. Several respondents argued that relevant government departments must increase their capital investment and talent pool.

“In fact, human and financial investment is not enough. Our hospital actually has an emergency team, but each member has much daily work to do, and sometimes they do both. If we encounter a public health emergency, human resources are necessarily not enough” (P4).

## Discussion

### Carry out emergency continuing education training to cultivate professional talents

Generally, professional emergency personnel with solid theoretical knowledge and rich practical experience capabilities are scarce in China (52). Previous studies have indicated that the emergency rescue abilities of clinical medical workers, community medical workers, rescue team members, and other professionals in public health emergencies in China are average to low (53, 54). Thus, they are often unable to meet the needs of emergency rescue in public health emergencies, and this, to some extent, limits improvements in emergency rescue quality. The results show that most participants in the study have a high demand for continuing education and training in emergency rescue and management. Emergency rescue in public health emergencies requires not only first aid knowledge and skills but also epidemiological knowledge, risk assessment abilities, and public health response abilities (26, 55).

The more educated a professional is, the more comprehensive their knowledge of the profession, and the more motivated and active they will be at work (56). However, the emergency management personnel involved in public health emergencies are generally not highly educated and have a low professional level. In China, for example, more than half (54%) of the personnel in China’s Centers for Disease Control and Prevention have only a college degree, about one-third (37%) have a Bachelor’s degree, and only 7% have a Master’s degree (57). Such educational levels suggest that relevant departments should carry out continuing education and training on emergency management and rescue for professionals with different levels, educational backgrounds, and positions, based on their different needs, with improved training contents and methods. The training content should not be limited to the theory and skill training of emergency rescue personnel. However, it must also provide training in preventive isolation, epidemiological monitoring, quarantine and disinfection, and psychological assistance. With the rapid development of information technology, applications such as WeChat, networks, and other platforms could be utilized to establish online learning courses so that training time is more mobile (58). Scientific retraining is the basis of relevant emergency skills retention. Such outcomes emphasize the importance of regular provision of continuing education and training for professionals involved in emergency management and rescue (59).

### Strengthen emergency drills and improve practical ability

Several participants in this study mentioned that, although they have a certain theoretical understanding of public health emergencies, they do not have much experience in public health emergencies. Therefore, when things do happen, they are still busy with their usual tasks and do not know how to deal with the emergency. One study found that those who regularly participated in emergency drills generally had higher emergency rescue capacities than those who did not participate in relevant drills (60). Through emergency drills, professionals can become familiar with the contents of emergency plans, work processes, and personal responsibilities in an emergency rescue through exposure to real scenes similar to public health emergencies. Such exposure can also increase their theoretical knowledge and build their confidence in participating in real rescue situations (61, 62). Therefore, in the daily training process, hospitals and relevant departments should not only provide theoretical education but must also regularly hold emergency drills for public health emergencies. Such drills would not only deepen the understanding and experience of professionals in dealing with public health emergencies but would also improve their crisis awareness and emergency response abilities. Through emergency drills, shortcomings and loopholes in the relevant emergency plans can be identified and adjustments can be made further to improve the institution’s emergency plan and responsibility.

### Make emergency plans and establish an emergency team

Emergency plans play a crucial role in responding to public health emergencies (41, 63). Governments and medical institutions at all levels (i.e., provinces, cities, and counties) should formulate corresponding plans and procedures for handling public health emergencies based on relevant emergency plans, laws, and regulations of the state in combination with their actual situation. In addition, as an essential part of emergency capability, an emergency rescue team plays an vital role in emergency rescues. Public health emergencies are often unpredictable, so agencies should immediately deploy emergency response teams who specialize in dealing with public health emergencies and unify their command to ensure timely organization and management of the emergency (64). Public health events are different from other emergencies; they often require a large number of medical professionals. Therefore, it is necessary to further strengthen medical systems, encourage and support medical workers, strengthen the training of rescue workers, simulate the natural environment during exercises, and improve the professionalism of emergency rescue teams.

### Increased government investment and improved treatment

Public health services are public welfare provided by the government to the entire population; they play a vital role in the prevention and control of various diseases (65). The findings mentioned above, and interview results suggest that low wages, a lack of attention, and a lack of human resources are dilemmas faced by emergency rescue systems, which is consistent with previous research (66). Some participants mentioned that many public health emergency professionals work part-time. In addition to medical-related work, they also undertake many jobs. Their salaries and treatment are not proportional to their workloads, and thus, their work enthusiasm is reduced. Many respondents felt that emergency rescue work is irrelevant to their work, and they, therefore, ignore learning relevant knowledge, which, in turn, affects their abilities. Therefore, it is suggested that the government should pay attention to the treatment of professional staff, adjust the proportion of personnel, clarify the work responsibilities of professionals, appropriately improve their salaries and treatment, and improve the social status of relevant personnel (67) to promote their enthusiasm and initiative, increase their attention to emergencies, and improve their emergency rescue abilities.

## Conclusion

In this study, semi-structured in-depth interviews were conducted with 15 clinical medical workers, community medical workers, and rescue team members to understand the composition and factors influencing the emergency rescue abilities of professionals in dealing with public health emergencies. Professionals require an extensive knowledge reserve as well as warning and evaluation, information submission, emergency response, self-protection, personal, coordination and cooperation, and health education abilities to participate in emergency rescue. Education, region, experience, hospital level, and insufficient human and financial investment will affect a professional’s emergency rescue ability. Therefore, it is necessary further to optimize the provision of emergency education and training and cultivate professional talents. Relevant agencies should formulate emergency plans, establish emergency teams, and strengthen their emergency drills to improve the rescue ability of their agency. In addition, attention should be paid to the construction of an emergency rescue team, the adjustment of the personnel ratio, the improvement of staff treatment, and the promotion of work enthusiasm to improve the emergency rescue abilities of professionals in dealing with public health emergencies. These authors thank the professionals participating in this study who shared their experiences with us.

## Data availability statement

The raw data supporting the conclusions of this article will be made available by the authors, without undue reservation.

## Ethics statement

The studies involving humans were approved by Ethical Review Board (ERB) of the Zhejiang Chinese Medical University. The studies were conducted in accordance with the local legislation and institutional requirements. The participants provided their written informed consent to participate in this study.

## Author contributions

FZ: Data curation, Formal analysis, Investigation, Writing – original draft, Writing – review & editing. YL: Data curation, Formal analysis, Investigation, Writing – review & editing. YC: Data curation, Formal analysis, Writing – review & editing. YG: Data curation, Formal analysis, Writing – review & editing. XZ: Conceptualization, Methodology, Resources, Supervision, Writing – review & editing.
